# Highly consistent genetic alterations in childhood adrenocortical tumours detected by comparative genomic hybridization

**DOI:** 10.1038/sj.bjc.6990691

**Published:** 1999-09

**Authors:** L A James, A M Kelsey, J M Birch, J M Varley

**Affiliations:** CRC Section of Molecular Genetics, Paterson Institute for Cancer Research, Wilmslow Road, Manchester, M20 9BX, UK; Department of Diagnostic & Molecular Paediatric Pathology CRC Paediatric & Familial Cancer Research Group, Royal Manchester Children's Hospital, Hospital Road, Pendlebury, Manchester, UK

**Keywords:** CGH, adrenocortical tumours, paediatric cancer

## Abstract

We have examined 11 cases of childhood adrenocortical tumours for copy number changes using comparative genomic hybridization (CGH). The changes seen are highly consistent between cases, and are independent of tumour type (carcinoma versus adenoma) or the presence of a germline *TP53* mutation. The regions of chromosomal gain and loss identified in this study indicate the location of genes that are potentially important in the development and progression of childhood adrenocortical tumours. Finally, the copy number changes identified in childhood tumours are distinctly different to those seen in adult cases (Kjellman et al (1996) *Cancer Res***56**: 4219–4223), and we propose that this indicates that childhood tumours are of embryonal origin. © 1999 Cancer Research Campaign


*British Journal of Cancer* (1999) **81**(2), 300–304
© 1999 Cancer Research Campaign
Article no. bjoc.1999.0691

### Highly consistent genetic alterations in childhood adrenocortical tumours detected by comparative genomic hybridization

**LA James[1], AM Kelsey[2], JM Birch[3] and JM Varley[1]**

[1]CRC Section of Molecular Genetics, Paterson Institute for Cancer Research, Wilmslow Road, Manchester, M20 9BX, UK; [2]Department of Diagnostic & Molecular Paediatric Pathology and [3]CRC Paediatric & Familial Cancer Research Group, Royal Manchester Children's Hospital, Hospital Road, Pendlebury, Manchester, UK

**Summary** We have examined 11 cases of childhood adrenocortical tumours for copy number changes using comparative genomic hybridization (CGH). The changes seen are highly consistent between cases, and are independent of tumour type (carcinoma versus adenoma) or the presence of a germline *TP53* mutation. The regions of chromosomal gain and loss identified in this study indicate the location of genes that are potentially important in the development and progression of childhood adrenocortical tumours. Finally, the copy number changes identified in childhood tumours are distinctly different to those seen in adult cases (Kjellman et al (1996) *Cancer Res* **56**: 4219–4223), and we propose that this indicates that childhood tumours are of embryonal origin.

© 1999 Cancer Research Campaign

**Keywords:** CGH; adrenocortical tumours; paediatric cancer

Adrenocortical carcinoma (ACC) is rare. Data from the Manchester Children's Tumour Registry (Birch, 1988) show that, in children aged less than 15 years, ACC occurs at a rate of approximately 0.3 per million person years, with a median age of onset of 3 years. ACC accounts for about 0.3% of all paediatric malignancies. Adult ACC constitutes 0.02% of all malignancies and shows a peak age of onset of around 60 years (data from Cancer Registries, England and Wales, 1987–1991, Office of National Statistics, London). Few data are available on the genetic analysis of adrenocortical tumours, although a recent report (Kjellman et al, 1996) identified a number of common regions of DNA imbalance across the genome in adult ACC using the technique of comparative genomic hybridization (CGH). In adult ACC, the changes detected included an increased relative copy number of chromosomes 4q, 5p and 5q and loss of chromosomes 2, 11q and 17p. Very few aberrations were detected in any of the adult adrenocortical adenomas examined.

In the present analysis we have applied CGH to a series of 11 adrenocortical tumours, two adenomas and nine carcinomas, from patients below the age of 15 years (mean 4.2 years). The results indicate a highly consistent pattern of DNA gains and losses across the genome in both histopathological subtypes that will allow further investigation of the precise genetic aberrations involved in this disease. Most interestingly, the changes seen in childhood adrenocortical tumours are largely distinct from those seen in adult cases, indicating that, although morphologically similar, they may represent different tumours, with childhood tumours possibly arising from the fetal adrenal cortex.

*Received 19 November 1998*
*Revised 5 February 1999*
*Accepted 16 February 1999*

*Correspondence to:* JM Varley

### MATERIALS AND METHODS

### Patient material

Specimens from 11 cases of childhood adrenocortical tumours (mean patient age 4.2 years, range 9 months–14 years) were examined. All tissues were fixed for a minimum of 24 h in buffered formalin (0.4% sodium acid phosphate, 0.65% sodium phosphate) prior to paraffin embedding. In all cases the grade and pathological features of the tumour were examined by one of us (AK). There are no definitive clinical histological criteria for malignancy in childhood adrenocortical tumours (Medeiros and Weiss, 1992). Tumours were classified as adrenocortical carcinoma (nine cases) or adenoma (two cases) on the basis of histopathological features including the number of mitoses, pleomorphism, invasion, necrosis and tumour size. Clinical outcome was also taken into consideration. The percentage of tumour cells in each sample exceeded 80%. Patient information and tumour details are given in Table 1. None of the patients had received treatment prior to surgical removal of their tumours.

### Comparative genomic hybridization

Test DNA was prepared from four sequential 10-µm sections removed from each paraffin-embedded tumour sample. These were dewaxed with octane, dehydrated using 100% ethanol and the specimen was then air-dried prior to overnight incubation at 37°C in 100-µl digestion buffer (50 mM Tris pH 8.5, 1 mM EDTA, 0.5% Tween-20) containing 20 µg proteinase K. The samples were heated at 95°C for 10 min, microfuged and the supernatant was removed to a clean tube for storage at –20°C and later use in degenerate oligonucleotide primed (DOP) polymerase chain reaction (PCR). Control DNA from a normal female placenta and DNA from one case of childhood adrenocortical carcinoma were prepared using standard methods (Sambrook et al, 1989).

**Table 1** Patient details and classification of adrenocortical tumours studied together with CGH results obtained.

| Case | Sex | Age at diagnosis (years) | Tumour classification | TP53 germline mutationa | CGH analysis Losses | Gains | Amplifications |
|---|---|---|---|---|---|---|---|
| 1 | F | 2 | Carcinoma | R175H | 6q16-q24, 18q12, X | 1p34.2-pter, 5q31-qter, 9q34, 19, 20p13, 20q | |
| 2 | F | 1 | Carcinoma | Y220C | 18q12–21 | 1p33-pter, 11q13, 16p11.2, 16q23-qter, 17, 21q, 22q | 9q34, 19 |
| 3 | F | < 1 | Carcinoma | R158H | 4p15.3–4q33, 11q22, 13q21–31, 18q11.2–21, X | 1p33-pter, 6p21.1-p21.3, 11q12–13, 12q23-qter, 15q22–23, 16, 22q | 7q36, 9q34, 19 |
| 4 | F | 3 | Carcinoma | nk | 2q12-q34, 3q26.2-qcen, 4p15.2-qter, 6q22-qcen, 9p13-pter, 11p12–14 | 1q21, 7q22-qcen, 11q12–13, 12q12–13, 15q22-qter, 17q21, 20q11.2, 21q22, 22q | 1p32-pter, 5q34-qter, 7p21-pter, 7q33-qter, 9q34, 17q24-qter, 19 |
| 5 | F | 14 | Carcinoma | P152L | 4q21-q26, 13q21–q31, 18q12–q22, Xp22.1-pcen, Xq21-q26 | 1p33-pter, 6p21.3, 7q36, 11q12–13, 12q24.1-qter, 17, 16p, 20q, 21q | 7p22, 9q34, 19, 22q |
| 6 | F | 4 | Adenoma | (P152L) | 3pter-q26.3, 4p15.3-q31.1, 11p14-pcen, 11q14-qter, 18q12–21 | 1p33-pter, 6p21.3, 15q22, 16p12-pcen, 22q | 9q34, 19 |
| 7 | F | 4 | Carcinoma | P152L | 2p22-pter, 2q14.1-q32, 5q11.2-q22, 8q22-q24.1, 11p14-pcen, 18q12–21, X | 1p31-pter, 6q, 7p21-pter, 7q11.2, 7q36, 9q, 12, 17q21 | 19 |
| 8 | M | 7 | Adenoma | none | 1p22, 2q21–32, 3p14–q13.2, 4p15.3-q32, 5p14-q23, 6q15-q22, 7p15, 13q12–31, 18q12–21, X | 6p21.3, 12q24.1-qter, 16q, 17, 20q | 1p32-pter, 7q36, 9q34, 11q12–13, 19, 22q |
| 9 | M | < 3 | Carcinoma | R158H | 6p22-pter | 1p36.1-pter, 7p22, 12pter-q13, 12q23-qter, 15, 19 | |
| 10 | F | < 2 | Carcinoma | R213Stop | 2, 4, 11, 13, 17, 18, 21q22 | 5, 9pter-q33, 12pter-q23, 16q22-qter, 19, 20, 22q | |
| 11 | F | 5 | Carcinoma | P152L | 3p22–q26.2, 4p15.3-q33, 11p14-pcen, 11q14-qter, 14q21-q24 | 1p32-pter, 1q21, 5q34-qter, 7q, 12q24.1-qter, 16p, 20q, 22q | 7p22, 9q34, 19 |

[a] The status of the patient with respect to a germline *TP53* mutation is shown where known. Patient 6 has an inferred germline mutation (Varley et al, manuscript submitted) and this is indicated by the site of the mutation given in parentheses.

Detailed methods for the preparation and labelling of probes from paraffin-embedded material and their use in CGH have been described elsewhere (James and Varley, 1996; James et al, 1997). Briefly, both test and control DNA were separately amplified and labelled using DOP-PCR (Telenius et al, 1992). The test DNA was labelled with fluorescein isothiocyanate (FITC)-11-dUTP (Fluoro-Green, Amersham) whilst the control DNA was labelled with Rhodamine-4-dUTP (Fluoro-Red, Amersham). Metaphase chromosome spreads were prepared from phytohaemagglutinin (PHA)-stimulated lymphocytes obtained from a cytogenetically normal, healthy male donor according to methods outlined previously (Mitchell et al, 1995). Chromosomes were denatured in 70% deionized formamide 2 × sodium–saline citrate (SSC) at 80°C for 4 min and dehydrated through an ice-cold ethanol series immediately prior to use. Fluorescence in situ hybridization, microscopy and image analysis were performed exactly as described previously (James et al, 1997).

Each CGH experiment included a slide hybridized with equal quantities of normal control DNA separately labelled with FITC and rhodamine. This was used to set the fluorescence ratio thresholds later applied to the analysis of the adrenocortical samples. Only differences in the fluorescence ratio which fell outside the fluctuations seen in the normal control hybridization were taken as evidence of loss or gain of regions from the tumour DNA (see Results). The validity of the CGH data obtained was tested using a sample from patient 1. This DNA was amplified, labelled and employed in two separate CGH experiments and the results were compared.

### RESULTS

This study represents the first report of the cytogenetic analysis of childhood tumours of the adrenal cortex using CGH. In all cases except one, DNA was extracted from formalin-fixed, paraffin-embedded sections and all DNAs were amplified by DOP-PCR and labelled for CGH. Prior to the analysis of tumour samples, the control slide, hybridized with equal quantities of rhodamine- and FITC-labelled normal placental DNA, was examined to assess the quality of the data produced, and to set the fluorescence ratio thresholds for later analysis of the tumour data. An even hybridization was achieved across all chromosomes which resulted in a fluorescence profile averaged from at least six homologues which was close to 1.0 for each chromosome. Based on these data, an FITC:rhodamine ratio of greater than 1.15 was chosen to identify DNA copy number increases and of less than 0.80 to identify copy number decreases in the tumours. We further defined low level gain where the ratio increased above 1.15 and up to 2.0, and amplification where the ratio increased over 2.0.

The validity of the CGH data obtained in this study was confirmed by the analysis of data from two separate experiments using patient 1. Initial CGH results suggested a number of regions of DNA copy number change including losses on chromosome 6q and 18q and gains on 1p, 19 and 20. After re-amplification of the tumour DNA a second CGH experiment was performed. This identified exactly the same regions of DNA copy number change, which established the reproducibility of the technique and the validity of the data obtained.

© 1999 Cancer Research Campaign 

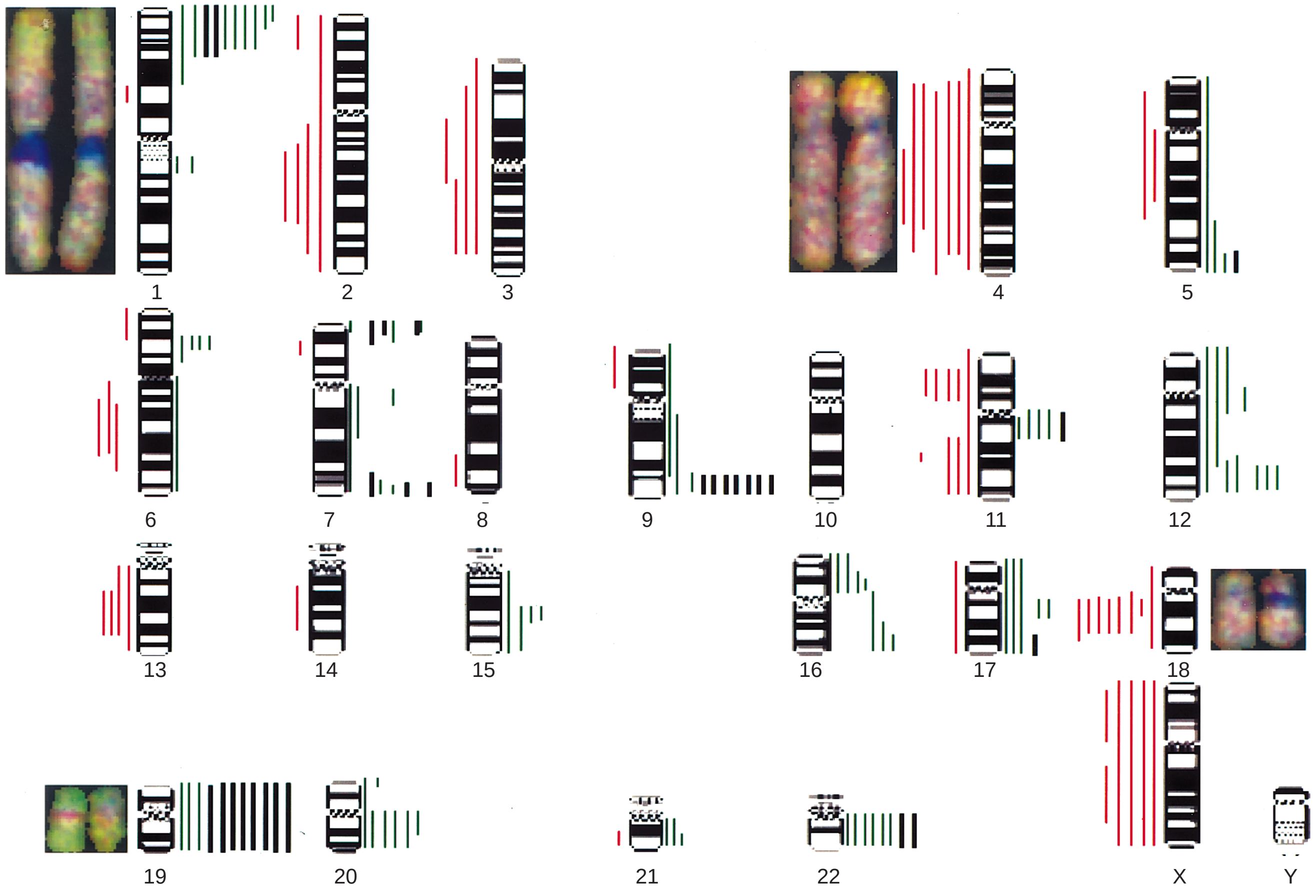

**Figure 1** Summary of the DNA copy number changes identified in eleven cases of childhood adrenocortical tumours by CGH. Red bars to the left of each chromosome represent copy number losses, and green and black bars to the right of each chromosome represent copy number gain and amplification respectively. Images of chromosomes 1, 4, 18 and 19 from patient 11 are shown

Table 1 and Figure 1 summarize the data obtained from each case examined. All of the samples, both those classified as adenoma and those as carcinoma, had multiple copy number aberrations (mean 14.5 per tumour, range 7–22). Notably, regions of increased copy number were identified on chromosome 19 in all 11 cases and 1p and 9q in 10/11 cases examined.

Other common regions of DNA gain were seen on chromosomes 22q (8/11), 12q (8/11), 11q (5/11), 17q (5/11) and 20q (6/11). The minimal common regions of gain were at 1p35-pter, 9q34, 12q24.1-qter, 17q21 and 11q13. The most frequently observed losses involved chromosomes 18q (8/11), 4 (7/11), X (5/11), 3 (4/11) and 2 (4/11). The minimal common regions of deletion were at 18q21, 4q21–q28, Xp22.2-pcen, Xq21–q25, 3cen-q13.2 and 2q21–q32. There were no significant differences between either the number or the type of copy number changes between adenomas and carcinomas (see Table 1).

Of the 11 cases of childhood adrenocortical tumours reported here, two have been previously described by our group as belonging to Li-Fraumeni syndrome families (patients 1 and 2, Table 1) (see Birch et al, 1994; Varley et al, 1995, 1997). In both of these cases germline *TP53* mutations have been identified. The remaining nine cases of childhood adrenocortical cancer were unselected for family history, but have all been screened for germline *TP53* mutations as part of a separate study (Varley et al, manuscript submitted). One case was shown to have no detectable germline mutation (patient 8), and we were unable to analyse constitutional DNA from one other. All the remainder had germline mutations as shown in Table 1. There are no obvious differences in the patterns of chromosomal gains and losses according to germline *TP53* mutation status.

### DISCUSSION

We report here a study of 11 cases of childhood adrenocortical tumours for copy number changes as identified by CGH. All cases showed multiple alterations, and there were a number of highly consistent chromosomal changes. There were no significant differences in the CGH karyotypes between adenomas and carcinomas, or between cases with and without germline *TP53* mutations.

The changes observed in the childhood tumours are shown diagramatically in Figure 1. A high proportion of the changes are highly consistent, with three regions showing copy number gains in over 90% of cases (1p36, 9q34 and chromosome 19). A recent review of DNA copy number amplification in human tumours (Knuutila et al, 1998) describes only one amplicon which is found at a comparable frequency in any tumour; the 12p11–12 amplicon in male germ cell tumours. In addition to the above, we report a number of other regions which show gain of copy number in over

 © 1999 Cancer Research Campaign

**Table 2** A comparison of the changes reported in a series of cases of adult adrenocortical tumours (*Kjellman et al*, 1996) and those described in this report in childhood adrenonocortical tumours

| Adult | | | Childhood | | |
|---|---|---|---|---|---|
| **Chromosome copy number gain/amplification** | | | | | |
| 4q | (4/8) | 4q31 | 1p | (10/11) | 1p36 |
| 5p | (4/8) | | | | |
| 5q | (4/8) | | | | |
| | | | 9q | (10/11) | 9q34 |
| | | | 11q | (5/11) | 11q13 |
| 12 | (3/8) | | 12q | (8/11) | 12q24.1-qter |
| 15q | (3/8) | 15q21-qter | | | |
| 16q | (3/8) | | | | |
| | | | 17q | (5/11) | 17q21 |
| 19p | (3/8) | | 19 | (11/11) | |
| | | | 20q | (6/11) | |
| | | | 22q | (8/11) | |
| **Chromosome copy number loss** | | | | | |
| 2 | (4/8) | 2p23-cen-q21 | 2 | (4/11) | 2q21-32 |
| 3p | (3/8) | 3p21-cen | 3 | (4/11) | 3cen-q13.2 |
| | | | 4 | (7/11) | 4q21-q28 |
| 6q | (3/8) | | | | |
| 8p | (3/8) | | | | |
| 9p | (3/8) | | | | |
| 11p | (3/8) | | | | |
| 11q | (4/8) | 11q22-qter | | | |
| 17p | (4/8) | | | | |
| 17q | (3/8) | | | | |
| 18q | (3/8) | | 18q | (8/11) | 18q21 |
| 22q | (3/8) | | X | (5/11) | Xp22.2-pcen |
| | | | | | Xq21-q25 |

The most frequently reported alterations in each study are listed. In each category the chromosome/arm involved is indicated, the frequency with which it is altered, and the minimal common regions of gain or loss if relevant.

50% of tumours (12q, 20q and 22q). Regions of loss are more limited in both number and consistency between cases, but nonetheless two areas show frequent loss (18q21 and 4q21–q28), with four other areas showing loss in around 50% of cases (2q21–q32, 3cen-q13.2 and two regions of the X chromosome).

A number of the chromosomal regions that we have identified in this study have been reported by others to be implicated in a variety of malignancies. For example, gain/amplification of 11q13, 17q21, regions of chromosome 19 and 20q have all been reported in a number of solid tumours (reviewed in Knuutila et al, 1998). Similarly, losses of chromosome 18 have been described in breast, colorectal, lung, pancreatic and head and neck tumours. Candidate genes have been identified in some of these regions, for example *CCND1* and *INT2* at 11q13, *c-erbB2* at 17q21 and *DCC* at 18q21. However, the pattern of copy number aberrations reported here in childhood adrenocortical tumours is unusual, and most closely resembles that reported in oral squamous cell carcinomas (Wolff et al, 1998). There is no obvious biological significance to this observation. Immunohistochemical staining for cyclin D1 confirmed that there is overexpression in the majority of the adrenocortical tumours, including a number with no apparent gain at 11q13 (data not shown), implicating cyclin D1 in the development or progression of childhood adrenocortical tumours.

Chromosome band 9q34, shows gain/amplification in 10/11 tumours reported here, and the cellular oncogene *c-abl* maps to this region, making it a possible target for the copy number gain seen. However also mapping within the same region is a gene whose function is essential for both the development and the function of the adrenal cortex, *SF-1* (Taketo et al, 1995). This gene encodes steroidogenic factor 1 (SF-1) which is detected at the earliest stages in the development of the urogenital ridge, from which the precursors of both the adrenal cortex and the gonads derive (reviewed in Parker and Schimmer, 1997). SF-1 continues to be expressed in the cells of the adrenal cortex throughout embryonic and adult life and regulates the co-ordinate expression of a number of genes in the adrenal cortex, including cytochrome P450 steroid hydroxylases and β-HSD. We are currently examining the possibility that *SF-1* could be the target gene in the amplicon at 9q34.

The differences between adult and childhood adrenocortical tumours with respect to their patterns of chromosomal gain and loss are very striking. Table 2 lists the chromosomal regions that show the most frequent copy number aberrations in adult (Kjellman et al, 1996) and childhood tumours (this study). It is immediately apparent that the patterns are very different, and almost mutually exclusive. The only regions that show frequent alterations in common to both types are regions of chromosome 19 and 18q. This observed difference clearly demonstrates that the genetic changes associated with the development and progression of adult and childhood adrenocortical tumours are quite distinct. This idea is further supported by the apparent bimodal age distribution of childhood and adult disease. Finally, childhood

© 1999 Cancer Research Campaign 

adrenocortical tumours are strongly associated with germline *TP53* mutations (Wagner et al, 1994; Varley et al, manuscript submitted) unlike adult disease.

We propose that childhood adrenocortical tumours (both carcinomas and adenomas) are embryonal tumours, derived from fetal adrenal cortex, in contrast to adult-onset tumours that arise from adult cortex. A proportion of childhood adrenocortical tumours produce cortisol although in vivo cells of the fetal zone do not produce β-HSD, a key enzyme in the production of cortisol (reviewed in Mesiano and Jaffe, 1997*a*). This may appear to contradict our hypothesis that childhood adrenocortical tumours arise in the fetal cells. However, in vitro fetal zone cells can produce β-HSD when treated with ACTH, and the levels of a variety of growth hormones and their receptors can influence both the levels of expression of ACTH in the fetal pituitary and its receptor on fetal zone cells of the adrenal gland (see Mesiano and Jaffe, 1997*b*). It is possible that inherited defects in *TP53* result in failure of the complete programmed apoptosis of the fetal cortex that occurs around birth (Mesiano and Jaffe, 1997*a*). This, coupled with an increase in genomic instability consequent on the presence of a mutant p53 protein, leads to a vastly elevated risk of adrenocortical tumours in children with germline mutations. The copy number aberrations that we have described in childhood tumours would therefore reflect genetic alterations specific to the transformation of fetal, rather than adult, adrenal cortex.

### NOTE ADDED IN PROOF

Following acceptance of this manuscript, Figueiredo et al (1999) (*J Clin Endocrinol Metab* **84**: 1116–1121) published a CGH analysis of nine cases of non-familial childhood ACC which identified a similar pattern of DNA gain and loss.

### REFERENCES

Birch JM (1988) Manchester Children’s Tumour Registry 1954–1970 and 1971–1983. In: *International Incidence of Childhood Cancer*, Parkin DM, Bieber A, Draper GJ, Stiller CA, Terracini B and Young JA (eds). IARC Scientific Publications No 87, IARC: Lyon

Birch JM, Hartley AL, Tricker KJ, Prosser J, Condie A, Kelsey AM, Harris M, Morris Jones PH, Binchy A, Crowther D, Craft AW, Eden OB, Evans DGR, Thompson E, Mann JR, Martin J, Mitchell ELD and Santibanez-Koref MF (1994) Prevalence and diversity of constitutional mutations in the *p53* gene among 21 Li–Fraumeni families. *Cancer Res* **54**: 1298–1304

James L and Varley J (1996) Preparation, labelling and detection of DNA from archival tissue sections suitable for comparative genomic hybridisation. *Chromosome Res* **4**: 163–164

James LA, Mitchell ELD, Menasce L and Varley JM (1997) Comparative genomic hybridisation of ductal carcinoma in situ of the breast: identification of regions of DNA amplification and deletion in common with invasive breast cancer. *Oncogene*, **14**: 1059–1066

Kjellman M, Kallioniemi O-P, Karhu R, Höög A, Farnebo L-O, Auer G, Larsson C and Bäckdal M (1996) Genetic aberrations in adrenocortical tumors detected using comparative genomic hybridization correlate with tumor size and malignancy. *Cancer Res* **56**: 4219–4223

Knuutila S, Bjorkqvist A-M, Autio K, Tarkkanen M, Wolf M, Monni O, Szymanska J, Larramendy ML, Tapper J, Pere H, El-Rifai W, Hemmer S, Wasenius V-M, Vidgren V and Zhu Y (1998) DNA copy number amplifications in human neoplasms. *Am J Pathol* **152**: 1107–1123

Medeiros LJ and Weiss LM (1992) New developments in the pathologic diagnosis of adrenal cortical neoplasms: a review. *Am J Clin Pathol* **97**: 73–83

Mesiano S and Jaffe RB (1997*a*) Developmental and functional biology of the primate fetal adrenal cortex. *Endocrine Rev* **18**: 378–403

Mesiano S and Jaffe RB (1997*b*) Role of growth factors in the developmental regulation of the human fetal adrenal cortex. *Steroids* **62**: 62–72

Mitchell ELD, Jones D, White GRM, Varley JM and Santibanez-Koref MF (1995) Determination of the gene order of three loci, CD2, NGFβ, and NRAS at human chromosome band 1p13 and refinement of their localisation at the sub-band level by fluorescence in situ hybridisation. *Cytogenet Cell Genet* **70**: 183–185

Parker KL and Schimmer BP (1997) Steroidogenic factor 1: a key determinant of endocrine development and function. *Endocrine Rev* **18**: 361–377

Sambrook J, Fritsch EF and Maniatis T (1989) *Molecular Cloning. A Laboratory Manual*, 2nd edn. Cold Spring Harbor Laboratory Press: New York

Taketo M, Parker KL, Howard TA, Tsukiyama T, Wong M, Niwa O, Morton CC, Miron PM and Seldin MF (1995) Homologs of *Drosophila fushi-tarazu* factor 1 map to mouse chromosome 2 and human chromosome 9q33. *Genomics* **25**: 565–567

Telenius H, Carter NP, Bebb CE, Nordensköld M, Ponder BAJ and Tunnacliffe A (1992) Degenerate oligonucleotide-primed PCR: general amplification of target DNA by a single degenerate primer. *Genomics* **13**: 718–725

Varley JM, McGown G, Thorncroft M, Tricker KJ, Teare MD, Santibanez-Koref MF, Houlston RS, Martin J, Birch JM and Evans DGR (1995) An extended Li Fraumeni kindred with gastric carcinoma and a codon 175 mutation in *TP53*. *J Med Genet* **32**: 946–950

Varley JM, McGown G, Thorncroft M, Santibanez-Koref MF, Kelsey AM, Tricker KJ, Evans DGR and Birch JM (1997) Germ-line mutations of *TP53* in Li–Fraumeni families: an extended study of 39 families. *Cancer Res* **57**: 3245–3252

Wagner J, Portwine C, Rabin K, Leclerc J-M, Narod SA and Malkin D (1994) High frequency of germline p53 mutations in childhood adrenocortical cancer. *J Natl Cancer Inst* **86**: 1707–1710

Wolff E, Girod S, Liehr T, Vorderwulbecke U, Ries J, Steininger H and Gebhart E (1998) Oral squamous cell carcinomas are characterised by a rather uniform pattern of genomic imbalances detected by comparative genomic hybridisation. *Oral Oncol* **34**: 186–190

© 1999 Cancer Research Campaign

## References

[PDF_00356] Birch J. M., Hartley A. L., Tricker K. J., Prosser J., Condie A., Kelsey A. M., Harris M., Jones P. H., Binchy A., Crowther D. (1994). Prevalence and diversity of constitutional mutations in the p53 gene among 21 Li-Fraumeni families.. Cancer Res.

[PDF_00347] Figueiredo B. C., Stratakis C. A., Sandrini R., DeLacerda L., Pianovsky M. A., Giatzakis C., Young H. M., Haddad B. R. (1999). Comparative genomic hybridization analysis of adrenocortical tumors of childhood.. J Clin Endocrinol Metab.

[PDF_00364] James L. A., Mitchell E. L., Menasce L., Varley J. M. (1997). Comparative genomic hybridisation of ductal carcinoma in situ of the breast: identification of regions of DNA amplification and deletion in common with invasive breast carcinoma.. Oncogene.

[PDF_00361] James L., Varley J. (1996). Preparation, labelling and detection of DNA from archival tissue sections suitable for comparative genomic hybridization.. Chromosome Res.

[PDF_00398] Jay M. (1995). Ophthalmic genetics: a genealogical guide to sources in England and Wales.. J Med Genet.

[PDF_00368] Kjellman M., Kallioniemi O. P., Karhu R., Hög A., Farnebo L. O., Auer G., Larsson C., Bäckdahl M. (1996). Genetic aberrations in adrenocortical tumors detected using comparative genomic hybridization correlate with tumor size and malignancy.. Cancer Res.

[PDF_00372] Knuutila S., Björkqvist A. M., Autio K., Tarkkanen M., Wolf M., Monni O., Szymanska J., Larramendy M. L., Tapper J., Pere H. (1998). DNA copy number amplifications in human neoplasms: review of comparative genomic hybridization studies.. Am J Pathol.

[PDF_00376] Medeiros L. J., Weiss L. M. (1992). New developments in the pathologic diagnosis of adrenal cortical neoplasms. A review.. Am J Clin Pathol.

[PDF_00378] Mesiano S., Jaffe R. B. (1997). Developmental and functional biology of the primate fetal adrenal cortex.. Endocr Rev.

[PDF_00380] Mesiano S., Jaffe R. B. (1997). Role of growth factors in the developmental regulation of the human fetal adrenal cortex.. Steroids.

[PDF_00382] Mitchell E. L., Jones D., White G. R., Varley J. M., Santibanez Koref M. F. (1995). Determination of the gene order of the three loci CD2, NGFB, and NRAS at human chromosome band 1p13 and refinement of their localisation at the subband level by fluorescence in situ hybridisation.. Cytogenet Cell Genet.

[PDF_00387] Parker K. L., Schimmer B. P. (1997). Steroidogenic factor 1: a key determinant of endocrine development and function.. Endocr Rev.

[PDF_00391] Taketo M., Parker K. L., Howard T. A., Tsukiyama T., Wong M., Niwa O., Morton C. C., Miron P. M., Seldin M. F. (1995). Homologs of Drosophila Fushi-Tarazu factor 1 map to mouse chromosome 2 and human chromosome 9q33.. Genomics.

[PDF_00395] Telenius H., Carter N. P., Bebb C. E., Nordenskjöld M., Ponder B. A., Tunnacliffe A. (1992). Degenerate oligonucleotide-primed PCR: general amplification of target DNA by a single degenerate primer.. Genomics.

[PDF_00402] Varley J. M., McGown G., Thorncroft M., Santibanez-Koref M. F., Kelsey A. M., Tricker K. J., Evans D. G., Birch J. M. (1997). Germ-line mutations of TP53 in Li-Fraumeni families: an extended study of 39 families.. Cancer Res.

[PDF_00406] Wagner J., Portwine C., Rabin K., Leclerc J. M., Narod S. A., Malkin D. (1994). High frequency of germline p53 mutations in childhood adrenocortical cancer.. J Natl Cancer Inst.

[PDF_00409] Wolff E., Girod S., Liehr T., Vorderwülbecke U., Ries J., Steininger H., Gebhart E. (1998). Oral squamous cell carcinomas are characterized by a rather uniform pattern of genomic imbalances detected by comparative genomic hybridisation.. Oral Oncol.

